# Electrosurgical unit: Iatrogenic injuries and medico-legal aspect. Italian legal rules, experience and article review

**DOI:** 10.1016/j.amsu.2020.12.031

**Published:** 2021-01-04

**Authors:** Patrizia Gualniera, Serena Scurria, Daniela Sapienza, Alessio Asmundo

**Affiliations:** Departmental Section of Legal Medicine, Department of Biomedical and Dental Sciences and Morphofunctional Imaging, University of Messina, A.O.U. “G. Martino” Via Consolare Valeria n. 1, 98124, Messina, Italy

**Keywords:** Electrosurgical injury, Professional liability

## Abstract

**Background:**

The use of the electrosurgical unit (ESU) is well-established in the surgical practice. The Authors, to better understand the genesis of injuries connected to the use of electrosurgical instruments, conducted an in-depth literature review pertaining to this topic.

**Materials and method:**

Using the most important medical databases, a research of experimental studies in the last 20 years was conducted.

**Results:**

The analysis of the mechanisms responsible for the lesions showed that high energy devices remain as the most common cause of injury. Adverse events are mainly given by thermal injuries; cases of electromagnetic interference are also described in patients with pacemakers or sacral nerve stimulator and spinal stimulators as well as cases of fire of the endotracheal tube in the course of tracheostomy for the use of the electrosurgical unit in an environment with a high concentration of oxygen or anesthetic gases. Also reported in the literature are individual cases of fires caused by sparks from the electrosurgical handpiece also for the use of disinfectants and/or in relation to surgical drapes.

**Conclusion:**

In order to clearly define the medical-legal aspects, focusing on the professional responsibility of the surgical and nursing staff, the authors’ attention was brought to the need for an effective prevention plan that highlights not only the importance of an accurate procedural knowledge in order to safety use the electrosurgical instruments, but also the need for a system that monitors any complications or adverse events resulting from the use of such instruments.

## Highlights

• The use of the electrosurgical unit (ESU) is consolidated in surgical practice.
• Adverse events are mostly represented in electrosurgery.
• Literature in last 20 years describe than 5256 article with topic about iatrogenic damage in electrosurgery damage.
• Medico-legal features are important for the assessment of health workers liability.
• Improve prevention systems is the best solving in order to limit iatrogenic damage and consequently possible claims.

## 1 Introduction

The use of the electrosurgical unit (ESU) is well-established in the surgical practice thanks to the interaction between the cutting mechanism and the coagulative one. In Italy, according to article 1 of the legislative decree 46/97 and subsequent modifications, medical devices consist of: " … any instrument, apparatus, plant, substance or other product, used alone or in combination, including the computer software required for its proper functioning and aimed to be used in humans to diagnose, prevent, control, cure or mitigate of diseases … “. According to article 8 of the same legislative decree, the devices falls into classes I, IIa, IIb, III. This classification stems from the risk arising from the device use, on the basius of the following features: duration, invasiveness (non-invasive, invasive and surgically invasive), activity (an active medical device is “dependent, for its operation on a source of electric energy or of another kind of energy, different from that generated directly by the human body or by gravity and which acts by converting that energy ").

To better understand the genesis of electrosurgical injuries, it is convenient to recall that eletric energy interacts with human tissues which prove to be moderately conductive; the conductivity varies according to their water content, resulting lower for dry and high impedance tissues, and higher for wet and low impedance tissues. However, conductive differences are always very modest and such to allow the various electrosurgical techniques use standardization.

The current used in surgery is the high frequency alternating one (with a frequency higher than 300 kHz [1]) thanks to its feature to cause such rapid oscillations in the tissue ions that, apart from the production of heat, no other effect is generated: therefore, it allows to exploit the thermal effect of electric energy (the so called Joule effect) without the inconveniences of the faradic one which, due to the neuromuscular stimulation determined by the modification of the ion exchange physiological process, would cause spasms which may regard the heart fibrocell as well and that, therefore, might determine extrasistoles and even ventricular fibrillation [2].

Cutting and coagulation mechanisms are related both to current power - which ultimately generates heat - and to impulse modulation (i.e. to periodic interruptions of current), which are both influenced by the blood flow of the target tissue (which contributes to heat dissipation) and by the electrode dimension, in consideration of the fact that heat production is inversely proportional to the conductor section and that only in its vicinity a heat capable of generating such mechanisms can be reached [[3], [4], [5]].

The cutting action occurs due to the cell membrane rupture caused by the rapid water evaporation induced by the heat inside the cells which ultimately causes a solution of the adjacent tissue continuity. Water vapour develops near the electrode and then spreads, forming a kind of veil, between the conductor and the tissue: this allows a further spreading of the current, which triggers a chain reaction headed toward the working directions of the device and, at the same time, limits its dispersion to adjacent tissues. Naturally, the current intensity has to be enough to make the cells burst and it is therefore necessary that the temperatures reached all around the electrode are higher than 100 °C [3,6,7].

On the other hand, hemostasis occurs thanks both to the rapid protein coagulation and to a slow and synchronous water evaporation, leading to a cell aggregation with a “welding” effect which ultimately stops the bleeding. The coagulative effect occurs only if in the region where the electrode is in touch with the tissue temperatures of about 70°–80° are developed [3,5,6]. To optimize the coagulation mechanism, the current emissions must be extremely short in order to obtain a localized lesion, without damaging the surrounding tissues; a too high power would also cause a carbonization of the tissues with loss of conductivity, up to prevent the further propagation of heat [3,5,6].

Differently from the cutting mechanism (desiccation), it is modulation which makes the electrosurgical unit more suitable to the coagulative mechanism. Nonetheless, it is possible to achieve both the cutting and the hemostasis mechanisms, through the combination of their basic effects (i.e. blended mode) [7].

An electrosurgical circuit includes an electrosurgical unit (ESU), an active electrode, a patient and a dispersive electrode. The electric energy is supplied by the main power supplier and then it is converted to high frequency by a generator. It goes through the tissues thanks to metal electrodes with two distinct mechanism which characterizes, respectively, the monopolar and bipolar techniques and which are well-known and widely discussed both in literature and in the manuals issued by ESU system manufacturers [2,[5], [6], [7], [8]].

In the monopolar technique there is an active electrode - usually consisting of a small variously-shaped steel point - which is positioned on a handle and a neutral electrode (return pad) which closes the electric circuit allowing the energy to return to the generator without dispersions which might cause thermal lesions to the patient. the return pad is usually positioned in well dried body regions (human body resistance to electric current, which is usually of about 16,000 to 100,000 Ohms, in wet conditions lowers to 1000 Ohms, allowing current to go through the tissues much more easily and, therefore, this occurs when the skin is sweaty or wet or hairless), preferably on plane surfaces, possibly on wide muscular masses (being muscular tissue more conductive than fatty or bony one) and not very far from the operation field. the current path within the body has to be as short as possible, following an oblique direction and never a transversal one. The electrode is usually applied to the lumbar region in neck, chest and arm surgery, to the gluteal regions in gynecological and coxofemoral orthopaedic surgery, and to the back surface of thighs in urinary system surgery.

The bipolar technique is based on the electricity flow between two electrodes (an active and a neutral one) both positioned on the same handle and whose proximity, when the tissue is between them, allows to close the circuit. The bipolar electrodes, which are usually made up of forceps, are mainly used when an extremely accurate hemostasis is required in order not to damage nearby tissues and, therefore, above all in microsurgery or when important vascular and nervous structures are really close to the surgery area, and, therefore, it is widely used in neuro- and ophtalmological surgery.

Personal observation of some ESU thermal injuries and literature review lead us to investigate the related medico-legal issues wich mainly concern the professional responsibility of the health care workers. So, in order to better understand the genesis of lesions related to the use of electrosurgical instruments, the aim of this work was to conduct a review of the international literature relating to the aforementioned complications to evaluate their characteristics and extent and any corrective measures in the field of health professional liability.

## 2 Materials and method

Cochrane Database of Systematic Reviews, OVID MEDLINE, ScienceDirect, Web of Science, Pubmed and Scopus databases were used to search for experimental studies conducted in the last 20 years. The search was carried out using the following keywords: electrosurgery unit (or) electroscalpel (or) electrosurgery bipolar monopolar (and) electrosurgery injuries (and/or) electrosurgery errors (and) electrosurgery complication fire and sparks.

Than that 5256 papers were retrieved and these articles showed to be more relevant to the goals of the present article; the analysis was carried out according to the items included in the check list proposed and published in 2009, by the PRISMA group [9]. Inclusion and exclusion criteria, carried out according to PICO items [population = patient; intervention: electrosurgery; control: operative standard; Outcome: errors, complication and injuries], can be consulted in the supplementary information. The work has been reported in line with AMSTAR (Assessing the methodological quality of systematic reviews) Guidelines [10]. In table n. 1 articles with particular relevance (see Table 1).

**Table 1:** Articles with particular relevance.

1	Humes DJ, Ahmed I, Lobo DN. The pedicle effect and direct coupling: delayed thermal injuries to the bile duct after laparoscopic cholecystectomy. Arch Surg 2010;145:96–8
2	Choudhry AJ, Haddad NN, Khasawneh MA, Cullinane DC, Zielinski MD. Surgical Fires and Operative Burns: Lessons Learned From a 33-Year Review of Medical Litigation.Am J Surg 2017;213:558–564.
3	Alkatout I, Schollmeyer T, Hawaldar NA, Sharma N, Mettler L. Principles and safety measures of electrosurgery in laparoscopy. JSLS 2012;16:130–9.
4	Townsend NT, Jones EL, Paniccia A, Vandervelde J, McHenry JR, Robinson TN. Antenna coupling explains unintended thermal injury caused by common operating room monitoring devices. Surg Laparosc Endosc Percutan Tech 2015;25:111–13
5	Tixier F, Garçon M, Rochefort F, Corvaisier S. Insulation failure in electrosurgery instrumentation: a prospective evaluation. Surg Endosc 2016 Nov;30:4995–5001
6	Odell RC. Surgical complications specific to monopolar electrosurgical energy: engineering changes that have made electrosurgery safer. J Minim Invasive Gynecol 2013;20:288–98
7	Spruce L,Braswell ML. Implementing AORN Recommended Practices for Electrosurgery. AORN Journal 2012;95:373–86
8	Guideline Implementation: Energy-Generating Devices, Part 1dElectrosurgery 1.4 www.aornjournal.org/content/cme SHERYL P. EDER, MSN, RN, CNOR, CRCST
9	Sankaranarayanan G, Resapu RR, Jones DB, Schwaitzberg S, De S. Common uses and cited complications of energy in surgery. Surg Endosc. 2013;27:3056–72
10	Lipscomb GH, Givens VM. Preventing Electrosurgical Energy–Related Injuries. Obstet Gynecol Clin N Am 2010;37:369–77

## 3 Results

According to the literature high energy devices remain as the most common cause of injury. Understanding and addressing pitfalls in operative care may mitigate errors and potentially lessen future liability [11].

From consulted, injuries and equipment loss resulting from diathermy use are much more common than what we might think [12]. It is currently estimated that around 500 to 600 surgical fires occur annually in the United States [13].

Adverse events are mainly given by thermal injuries [6,7,[14], [15], [16], [17], [18]], which are more often related to an improper application of the neutral electrode and less frequently to unintentional contact of the active electrode with the tissue to dispersion phenomena during the use or to “insulation failure”, “direct coupling” and “capacitive coupling” [[19], [20], [21], [22], [23]].

Cases of electromagnetic interference are also described in patients with pacemakers [7,[24], [25], [26], [27]] or sacral nerve stimulator and spinal stimulators [28] as well as cases of fire of the endotracheal tube in the course of tracheostomy [7,[29], [30], [31], [32]] for the use of the electrosurgical unit in an environment with a high concentration of oxygen or anesthetic gases [33,34].

Also reported in the literature are individual cases of fires caused by sparks from the electrosurgical handpiece also for the use of disinfectants and/or in relation to surgical drapes [35].

The occurrence of thermal injuries, from data base of literature consulted, - would presumably imply the negligent responsibility of health workers. [36].

In this regard, studies conducted by Harder [37] on a series of complaints to insurance companies and judgments of the German judiciary have shown that in no case were these injuries considered to be justifiable, as foreseeable and avoidable events. The same literature underlines that such events are to be considered consequent to the lack of knowledge of the principles and bases of electrosurgery and therefore of the incorrect use of devices [[38], [39], [40], [41], [42]].

In order to avoid ESU burn related risks, it is first and foremost required an educational and continuous updating programme for the health staff in charge of the ESU, including the operating room nurses who will autonomously position the neutral pad and make sure that there are no malfunctions of the unit before every surgical operation.

The health staff should have sufficient technical knowledge of both the physics principles regulating the ESU functioning and of the adverse events related to its improper use or to its malfunctioning. [43]. Furthermore, it is required that the above mentioned staff strictly stick to the procedure required for a correct use of the unit in relation to the surgical operations to be performed and to the main safety and adverse event prevention regulations which are clearly stated in the guidelines [44].

In addition, since a patient undergoing an operation or another invasive procedure is under anesthetic and, most of the times, cannot guard himself or herself against any harm, the perioperative nurse's role in the planning, coordination, safe delivery and evaluation of the nursing care for the patient includes protecting him or her from the adverse effects of an energy generating device improper use.

The main measures to avoid adverse events during the use of the electrosurgical unit [[5], [6], [7], [41], [42], [45], [46]] consist therefore in:

- checking before every operation the cable and contact conditions as well as the alarm system
- a suitable preparation of the operating field avoiding flammable disinfectants
- choosing a neutral pad suitable to the required power
- positioning the neutral pad as close as possible to the operating field and in a way suitable to grant full adherence to the skin (hair removal in the region of interest, lack of bony prominences, skin folds and liquids), preferably applying a conductive gel between the skin and the pad
- lack of contacts between the neutral pad and the grounded mass
- positioning the monitoring device electrodes as far as possible from the operating field
- keeping current intensity as low as possible
- lack of contact between the patient's body and the metal parts of the operating table
- lack of contacts between the active electrode and the conductive parts of the operating table
- lack of unintentional contacts between the active electrode and the patient's body beyond the operating field.

## 4 Discussion and conclusions

When responsibility has to be ascertained, it is obvious that the expert's report should aim at finding to which negligent behavior the lesions caused by an electrosurgical unit may be attributed and, notably, if the responsibility of an adverse event causing a lesion to a patient may be ascribable to the surgeon, to the operating room nurse or to both.

As to this point, we believe that the burns caused by a current dissipation due to an improper positioning of the neutral electrode are mostly referable to a negligent liability of the operating room nurse. In fact, although in compliance with the surgeon's precise and detailed instructions, the nurse carries out the operating room procedures autonomously and is legally obliged to correctly fulfill his own duties, which include the positioning of the plate.

On the other hand, should the burns be the consequence of dissipation phenomena caused by an activated electrode, the nurse's behavior could hardly be considered negligent, unless there is a handle isolation defect, which is detectable in the preliminary phases. In such cases, the surgeon would be solely responsible for negligence in not having avoided such events, unless he can prove it was practically impossible to find them out since they could only be detected by the technical inspections arranged by the company he is working for.

Therefore, in evaluating negligent liability, besides a forensic medical assessment, it is also required an appraisal by technical experts who are capable of testing the real conditions of the devices and stating if their prospective anomalies could have been found or suspected by the health workers who used them. Such an evaluation is particularly complex and sometimes it is difficult or even impossible to carry out. The medical record should clearly report the presence or absence of generator induced tissue damage as well as well as the condition of the skin before the application and after the removal of the pad together with the location of the electrosurgical dispersive pad”. Further records might allow the traceability of the generator and its serial or ID number, although their presence is no longer recommended in the current guidelines. [47].

In the Italian legal system, according to the criminal law, the matter relates to the constitutional principle of personal liability, whilst as far as civil law is concerned, it deals with the compensation for damages. In the latter hypothesis, it is mainly the company to be involved and, at a later time, it can make up for the money loss at expenses of health-care workers (i.e., the surgeon, the nurse, the unit control responsible) who are considered liable for the lesions caused by the electrosurgical unit.

As to the damage evaluation, it must be considered that burn lesions can penetrate much farther than cutis since they can affect deeper structures and are even capable of interesting mucles, tendons, bones, nerves and blood vessels. Furthermore when vessels are involved due to local flow disturbances, the healing process can take longer and it is not rarely complicated by infections [46] so much so that sometimes it could be necessary to excise the eschar and, in case, to skin graft the area, exposing it to the risk of keloids, which may cause permanent outcome to be more serious.

We observed a patient who suffered from benign prostate hypertrophy and who, following an improper positioning of the neutral electrode, reported third-degree burns to postero-medial surface of the proximal third of the right leg and to the ipsilateral popliteal fossa pillars. There also occurred a thrombosis of the external saphenous vein and a partial involvement of the external popliteal sciatic nerve. Once the lesions were distinctly demarcated, the patient underwent a skin graft plastic surgery but, nonetheless, an electromyography administered at a later time showed that there were still signs of neural suffering in the territory of the above mentioned nerve.

It follows that also the consequences referable to the burn phenomena can become disabling not only for the changes they cause to skin and appearance but also to more complex functional activities (such as the neurological and circulatory ones) of the affected body regions.

Considering the relevant iatrogenic harmfulness of the electrosurgical unit, it is obvious that only an effective prevention will be able to limit the damages, which, as above mentioned, might be severe.

To reduce as much as possible the risk hypotheses, it is also necessary to check the units even before their marketing, to set up accurate and efficient information systems (e.g. the CE brand) available to users and organizations and to adopt adequate signaling systems which are able to report the accident occurred while using or monitoring the units.

Clinical risk management is conceived in the perspective of preventing damages to patients. It consists in defining company policies and in making sure that they are applied in order to set up and maintain the welfare safety through a definite organizational structure, which requires to consider effective risk predictors, with the perspective of the welfare quality continual improvement needed to give proof of professional excellence.

In such a context, drawing up the OR procedures cannot help considering both the iatrogenic potential of an electrosurgical unit and, accordingly, the need of detailed rules of behavior the health workers have to follow in order to avoid that such potential might turn into a real damage to the patient. The safe use of electrosurgical devices in the OR requires a multidisciplinary approach involving all the members of the surgical team as well as personnel from other departments such as the biomedical engineering and the risk management ones [44].

## Declaration of competing interest

The Authors confirm that there are no conflicts of interest associated with this publication.

## Research Registration Unique Identifying Number (UIN)

Please enter the name of the registry, the hyperlink to the registration and the unique identifying number of the study. You can register your research at http://www.researchregistry.com to obtain your UIN if you have not already registered your study. This is mandatory for human studies only.

1.Name of the registry:
2.Unique Identifying number or registration ID:
3.Hyperlink to the registration (must be publicly accessible):
  NOT registration needed for THIS ARTICLE review

## Availability of data and material

Yes.
