# Supplementary material for: Electrosurgical unit: Iatrogenic injuries and medico-legal aspect. Italian legal rules, experience and article review
Source: Ann Med Surg (Lond). 2021 Jan 4;62:26–30. doi: 10.1016/j.amsu.2020.12.031 (PMC7808912; doi:10.1016/j.amsu.2020.12.031)
Supplement: Multimedia component 1 [file mmc1.doc]

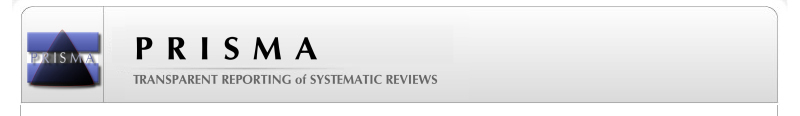
**PRISMA 2009 Flow Diagram**

**Screening**

**Included**

**Eligibility**

**Identification**

Records identified through database searching
(n = 5236)

Additional records identified through other sources
(n = 20 )

Records after duplicates removed
(n =3856)

Records screened
(n =1126)

Records excluded
(n =2730)

Full-text articles assessed for eligibility
(n = 366 )

Full-text articles excluded, with reasons
(n = 241)

Studies included in qualitative synthesis?
(n =47 )

Studies included in quantitative synthesis (meta-analysis)
(n = 47 )
